# Seroprevalence of *Toxoplasma gondii* in Pinnipeds under Human Care and in Wild Pinnipeds

**DOI:** 10.3390/pathogens10111415

**Published:** 2021-10-31

**Authors:** Micaela Martins, Nuno Urbani, Carla Flanagan, Ursula Siebert, Stephanie Gross, Jitender P. Dubey, Luís Cardoso, Ana Patrícia Lopes

**Affiliations:** 1 Zoomarine Portugal, 8201-864 Guia, Portugal; cat.crespo@hotmail.com (M.M.); nuno.urbani@zoomarine.pt (N.U.); carla.flanagan@zoomarine.pt (C.F.); 2 Institute for Terrestrial and Aquatic Wildlife Research, University of Veterinary Medicine Hannover, 25761 Buesum, Germany; ursula.siebert@tiho-hannover.de (U.S.); stephanie.gross@tiho-hannover.de (S.G.); 3 Animal Parasitic Diseases Laboratory, Beltsville Agricultural Research Center, Agricultural Research Service, US Department of Agriculture, Beltsville, MD 20705, USA; jitender.dubey@usda.gov; 4 Department of Veterinary Sciences and Animal and Veterinary Research Centre (CECAV), School of Agrarian and Veterinary Sciences, University of Trás-os-Montes e Alto Douro (UTAD), 5000-801 Vila Real, Portugal; aplopes@utad.pt

**Keywords:** antibodies, pinnipeds, Portugal, seroprevalence, modified agglutination test, *Toxoplasma gondii*

## Abstract

*Toxoplasma gondii* infection has been reported in numerous species of marine mammals, some of them with fatal consequences. A serosurvey for *T. gondii* infection was conducted in pinnipeds from an oceanographic park in Portugal (*n* = 60); stranded pinnipeds on the Portuguese coast (*n* = 10); and pinnipeds captured in Lorenzensplate, Germany (*n* = 99). Sera from 169 pinnipeds were tested for the presence of antibodies to *T. gondii* by the modified agglutination test with a cut-off titre of 25. An overall seroprevalence of 8.9% (95% confidence interval: 5.1–14.2) was observed. Antibody titres of 25, 50, 100, 1600 and ≥3200 were found in five (33.3%), two (13.3%), five (33.3%), one (6.7%) and two (13.3%) animals, respectively. Pinnipeds under human care had a seroprevalence of 20.0% (12/60), in contrast to 2.8% (3/109) in wild pinnipeds (*p* < 0.001). General results suggest a low exposure of wild pinnipeds to *T. gondii*, while the seroprevalence found in pinnipeds under human care highlights the importance of carrying out further studies. This is the first serological survey of *T. gondii* in pinnipeds in Portugal and the first infection report in South African fur seal (*Arctocephalus pusillus pusillus*).

## 1. Introduction

Toxoplasmosis is a worldwide zoonosis caused by the obligate intracellular parasite *Toxoplasma gondii*, which affects a wide range of homeothermic animals, including marine mammals. Despite the existence of several identified transmission routes, the exact mode of *T. gondii* transmission to marine mammals remains unknown [1]. The presence of felids, the definitive hosts, in nearshore environments can result in contamination of coastal habitats, because they are the only hosts that can excrete through their faeces, the environmentally resistant stages, the oocyst of *T. gondii*. Some sources of oocyst contamination include freshwater outflows to the sea, surface runoffs and litter from domestic cats flushed down toilets [1,2]. The ingestion of sporulated oocysts in marine environment, either directly or indirectly, is considered the main source of infection, in addition to ingestion of oocysts directly from marine waters or consumption of paratenic hosts (such as molluscs) that have concentrated the oocysts [3]. While *T. gondii* does not multiply in poikilothermic animals, bivalve molluscs, sardines and anchovies can assimilate and concentrate oocysts [4,5,6,7]. The consumption of infected birds flying over marine waters is another possible route of transmission [8]. Transplacental *T. gondii* transmission, although rare in pinnipeds, has been documented. Systemic toxoplasmosis was confirmed in a full-term stillborn Hawaiian monk seal (*Neomonachus schauinslandi*) pup and in aborted California sea lion foetuses (*Zalophus californianus*) [9,10]. Certain marine mammals, such as seals, also serve as food for humans [11]. Thus, *T. gondii* infections in marine mammals under human care and wild marine mammals are of clinical and public health importance [12]. Additionally, marine mammals are considered as sentinels of *T. gondii* contamination of the marine environment [1,11]. 

While numerous serological studies have been performed to determine the seroprevalence of antibodies to *T. gondii* in pinnipeds worldwide [13], to the best of our knowledge, there are no published reports of *T. gondii* infection in pinnipeds in Portugal. The purpose of this study was to perform a serological study on *T. gondii* infection in pinnipeds from the zoological collection of an oceanographic park (Zoomarine Portugal), as well as in pinnipeds stranded on the Portuguese coast and then rehabilitated at a rehabilitation centre for marine species (Porto d’Abrigo, Zoomarine Portugal), and in pinnipeds captured shortly for health assessment in Lorenzensplate, Wadden Sea, Germany.

## 2. Results

Antibodies to *T. gondii* were found in 15 (8.9%) of the 169 pinnipeds (95% confidence interval [CI]: 5.1–14.2) (Table 1). Titres of 25, 50, 100, 1600 and ≥3200 were found in five (33.3%), two (13.3%), five (33.3%), one (6.7%) and two (13.3%) of the seropositive animals, respectively. Antibodies to *T. gondii* were detected in five of the eight (62.5%) species tested, namely: Harbor seal (*Phoca vitulina*), California sea lion, South American sea lion (*Otaria flavescens*), South African fur seal (*Arctocephalus pusillus pusillus*) and hooded seal (*Cystophora cristata*).

Table 2 presents the seroprevalence of *T. gondii* in pinnipeds according to the independent variables studied. A significantly different seroprevalence (*p* < 0.001) was observed in pinnipeds under human care (12/60, 20.0%) compared with wild pinnipeds (3/109, 2.8%). In addition, there were statistically significant differences (*p* < 0.001) in frequencies of antibodies between otariids (10/37, 27.0%) and phocids (5/132, 3.8%). Concerning diet, pinnipeds fed fresh and frozen fish had lower seropositivity (3/109, 2.8%) than pinnipeds fed only frozen fish (12/60, 20.0%) (*p* < 0.001). 

The prevalence of antibodies to *T. gondii* was significantly different (*p* = 0.043) in pinnipeds under human care with clinical signs (4/8, 50.0%) compared with apparently healthy animals (8/52, 15.4%). On the other hand, the seroprevalence of *T. gondii* infection did not vary significantly in pinnipeds with respect to family (*p* = 0.107), sex (*p* = 0.508), age (*p* = 1.0), birthplace (*p* = 0.670), presence of felids around habitats, gestation (*p* = 0.340) and abortion (*p* = 1.0). 

With respect to paired samples, i.e., from the same individual, there were 11 out of the 12 seropositive pinnipeds under human care: Six were seropositive for both samples and five seronegative in the oldest sample (Table 3). 

In wild pinnipeds, statistically significant differences were not detected between the prevalence of infection and sex (*p* = 1.0), age (*p* = 0.421) and clinical status (*p* = 1.0).

## 3. Discussion

In the present study, the overall *T. gondii* seroprevalence (8.9%) was lower than in surveys elsewhere [11]. In the present study, most frequently observed titres in seropositive pinnipeds were low (titres of 25 and 100). These results are in agreement with other studies carried out in pinnipeds with the MAT [14,15,16,17,18,19,20,21].

The prevalence of antibodies to *T. gondii* detected in pinnipeds under human care was higher (20.0%) and significantly different from the one obtained in wild pinnipeds (2.8%), which may be explained by several factors, including the increased potential sources of infection in pinnipeds under human care. A likely source of infection is contamination of pool’s water source with sporulated oocysts, because there is a stray cat colony in the park, although managed through a veterinary care program that includes identification, vaccination, deworming and sterilization. In addition to direct exposure of water pool to feline faeces, pinnipeds could also be exposed to the oocysts mechanically transported through the trainer’s clothing or boots or even attending veterinarian, despite the use of specific footwear in areas where the animals are housed. The disinfection of swimming pool water in Zoomarine is carried out using chlorine and ozone treatments, but chlorination treatments (100 mg/L for 24 h) and ozone (6 mg/L for 12 min or 9.4 mg/L for 20 min) [22,23] may not destroy *T. gondii* oocysts [1]. As stated earlier, the ingestion of small birds with tissue cysts of *T. gondii* as a source of infection in seropositive animals cannot be ruled out. Arthropods and rodents may serve as mechanical vectors of *T. gondii*, but pest control is carried out regularly. The seroprevalence found in pinnipeds under human care in the present study (20.0%) was similar to the one found in South American sea lions from different Spanish zoological collections (1/8, 12.5%) [21] but considerably lower compared with the 50.0% (2/4) observed in California sea lions housed in a facility in Mexico [18] and the 72.7% (8/11) found in harbor seals from different Spain zoos [21]. 

The seroprevalence in wild animals reported in the present study suggests a low frequency of exposure to *T. gondii*. Comparatively, antibodies to *T. gondii* were not detected in any of the 120 harbor seals captured in Svalbard, Norway [24], neither in any of the 116 harbor seals in Glacier Bay and Kodiak Island, Alaska [25]. In north-eastern Atlantic harbor seals from the United Kingdom, a seroprevalence of 5.4% was reported [16]. Contrary to the results obtained in this study, substantially higher seroprevalences were reported in other investigations that also used the MAT, namely, in harbor seals from the Canadian Arctic (22.2%) [17] and the Scottish coast of the North Sea (29.0%) [26]. In grey seals, considerably higher seroprevalence was also reported, particularly from the Scottish coast of the North Sea (25.0%) and the Atlantic Ocean (40.0%) [26]. Despite the low seroprevalence obtained in the present study, the results indicate natural exposure to *T. gondii* in wild pinnipeds. These results may be explained by the low temperature of the waters that the seals included in this study inhabit, namely, the Wadden Sea and the North Atlantic Ocean, which may limit the survival of the oocysts [24]. Since the proximity to freshwater outflows to the sea is considered a risk factor [27], the seroprevalence found can be justified by poor contaminated near-shore environment. In addition, there may be different modes of transmission among species or different rates of exposure to the parasite, as some of the species, such as the hooded seal, perform seasonal migrations or extensive natural dispersal movements to southern waters, as far as Portugal, being exposed to hotter water currents [28].

Because among marine mammals, otariids spend more time on land than phocids, given the anatomical particularities that distinguish them [29], it is expected that the probability of exposure to the parasite will increase in individuals of the Otariidae family, because the permanency in a terrestrial environment, potentially contaminated with sporulated oocysts, is higher. Investigations in otariids are limited and seroprevalence varies depending on the species and the serological method used. For example, a 29.6% seroprevalence was found in 27 wild California sea lions from the coastal waters of southeast Alaska to the Bering Strait [14], using a cut-off MAT titre of 25. However, other studies report seroprevalences considerably lower. In Antarctic fur seals, only 2.4% (4/165) of the individuals were seropositive, also by using the MAT and a cut-off of 25 [19]. Similarly, only 2.5% of 1630 California sea lions had antibodies to *T. gondii* by indirect fluorescent antibody test (Carlson-Bremer et al., 2015). In New Zealand sea lions, only 6.0% (3/50) of the animals sampled were positive by latex agglutination test, using a cut-off titre of 32 [30].

The results obtained in phocids in the present study are in agreement with studies carried out in wild grey seals (5.8%) from France [31] and in Hawaiian monk seals (2.0%) [32]. In Japan, the observed seroprevalence in harbor seals was 4.0% (3/77) [33]. On the contrary, higher seroprevalences were found in Caspian seals in Iran (83.0%) [20] and in Antarctic Weddell seals (51.5%) [34]. In all of the investigations mentioned, the MAT was used as the serological test.

Concerning diet, and contrary to our expectations, a higher seroprevalence was observed in individuals fed exclusively on frozen fish. Considering that Zoomarine’s pinniped collection diet is based on thawed fish, previously frozen and stored at −20 °C for at least 21 days, it would be expected that the seroprevalence would be lower in these animals. However, it is important to remember that oocysts can survive freezing [1].

The higher seroprevalence obtained in pinnipeds under human care with clinical signs compared to that obtained in apparently healthy pinnipeds suggest that immunocompromised animals or animals with concomitant infections may be more prone to infection but serologic findings are only an aid to diagnosis of clinical toxoplasmosis [1].

Of the 60 pinnipeds under human care included in this study, antibodies to *T. gondii* were found in two harbor seals, two California sea lions, four South American sea lions and four South African fur seals. It should be noted that the lowest titre (i.e., 25), was found in harbor seals and California sea lions, while South African fur seals had the highest titres (i.e., 1600 and ≥3200). To the authors’ knowledge, this is the first study to report the presence of antibodies to *T. gondii* in South African fur seals. While seropositive South African fur seal with the highest titre (≥3200) had occasional clinical signs such as coughing, diarrhea and aggressiveness since its arrival at Zoomarine, some of which are observed in marine mammals with toxoplasmosis [3], antibodies are only an indicator of infection. 

Regarding paired samples, of 11 out of the 12 seropositive pinnipeds under human care, six were seropositive in both samples and five were seronegative in the oldest sample. These results suggest that in six animals the primary infection occurred in a place other than Zoomarine. However, the samples were not collected immediately after the animals entered the park and therefore it cannot be assumed that the infection did not happen at Zoomarine. On the contrary, five of those 11 seropositive animals tested negative in the old samples, suggesting that the infection occurred at Zoomarine. Of the 5 animals mentioned, one was the only seropositive pinniped born at Zoomarine. This pup probably acquired infection post-natally because its dam tested negative by MAT (data not shown). Additionally, vertical transmission of *T. gondii* in marine mammals is not frequent [24,33,35,36]. 

With respect to the antibody titre obtained in old and recent samples, we found that in three animals the titre decreased, in two it remained identical and in one it increased. The decrease in antibody titres in three animals over time is in agreement with the hypothesis that in general titres decrease over time [37]. In the remaining animals, the maintenance and even the increase of titres are suggestive of reinfection or continuous exposure to the agent [38].

The seropositive wild pinnipeds included two harbor seals (titres of 50 and ≥3200) captured in Lorenzensplate and one hooded seal (titre of 25) stranded on the south coast of Portugal. While the three species sampled have different eating habits, which may contribute to different exposure to the parasite, the results obtained may reflect permanency in contaminated coastal areas. Grey seals and hooded seals are species that spend more time out at sea, unlike e.g. harbor seals, thus reducing the likelihood of exposure to oocysts shed in coastal regions [16]. The observed differences between species may therefore be associated with lower exposure to the parasite in grey seals, especially if coastal oocyst runoff is considered the main source of exposure [2,16]. Since the susceptibility of pinniped species to infection by *T. gondii* is unknown, the natural resistance to this parasite may also explain the low seroprevalences obtained [9].

Because there are no drugs that eliminate *T. gondii* infection and the lack of a protective vaccine, prevention of infection by *T. gondii* acquires special importance, particularly in animals under human care. Hygiene measures, such as washing hands of trainers and veterinarians before and after contact with pinnipeds, as well as before and after handling fish, might reduce *T. gondii* transmission. Cats should not be allowed in or near the holding facility of marine mammals, and other agents such as arthropods that can constitute a means of dissemination of oocysts should be controlled.

## 4. Materials and Methods

### 4.1. Animals and Samples

In the present study, a total of 169 pinnipeds were tested, among which 60 were pinnipeds under human care and 109 were wild individuals (Table 4).

**Figure 1:**
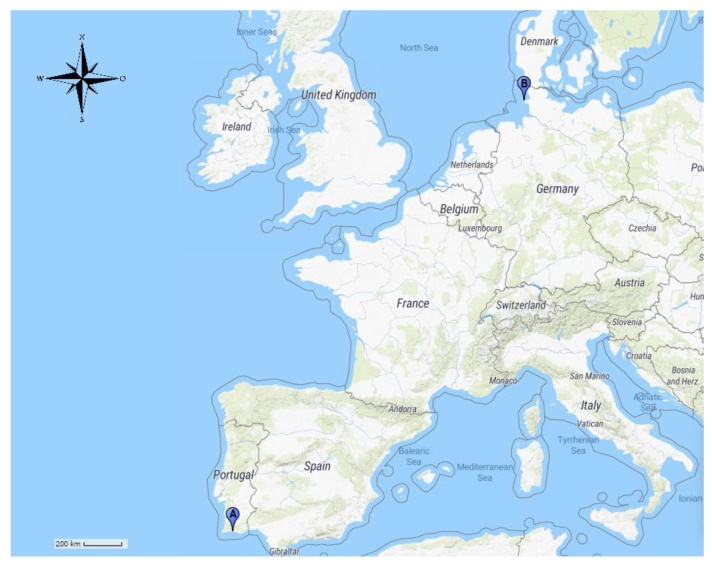
Location of Mundo Aquático SA - Zoomarine Portugal (**A**) and capture location of wild harbor seals included in the present study (**B**). Adapted from Scribble Maps [39].

Convenience-based sampling resulted in the collection of samples of seven different pinniped species from Mundo Aquático SA—Zoomarine Portugal, an oceanographic theme park located in the Algarve, south of mainland Portugal (Figure 1). The samples were collected between 1999 and 2020 during clinical procedures, with the vast majority of them having been obtained in the scope of a veterinary preventive medicine program. Additionally, and for some pinnipeds, it was possible to obtain a second sample, namely, the oldest sample, available in Zoomarine’s serum bank, obtained soon after birth or the arrival of the animals. This complementary assessment was made to determine in the seropositive animals whether the infection occurred at Zoomarine or elsewhere. Overall, the time window between the oldest and most recent sample ranged between 1 and 4 years.

Wild pinniped samples included hooded and grey seals stranded on the Portuguese coast, between 2001 and 2014, and were obtained after the animals had been admitted to Porto d’Abrigo—the Rehabilitation Centre for Marine Species of Zoomarine Portugal. Samples from harbor seals captured in Lorenzensplate, in the Wadden Sea, Germany (Figure 1) were also analysed. The samples were collected as part of the annual health monitoring program of the harbor seal population in the Wadden Sea of Schleswig-Holstein, between September 2014 and October 2019. The program is carried out by the Institute for Terrestrial and Aquatic Wildlife Research, part of the University of Veterinary Medicine Hannover, Foundation, Germany. For sample collection, the animals had been randomly selected. All animals were weight, measured, microchipped for tracking purposes, thus ensuring that no animal was sampled more than once, and subsequently released. 

In otariids, the blood was collected from the interdigital veins of the pelvic flippers with a 23 G butterfly catheter. In phocids, blood was collected from the extradural vein with 20 G needles or hind flippers (for details see [40]). In both cases, the collected blood was placed in dry tubes and subsequently centrifuged at 1500× *g* for 5 min. After separation, the serum of each sample was transferred to properly identified tubes and was then stored at −20 °C until analysis.

Whenever possible, epidemiological data were collected for each animal, namely: Sampling date, provenance (under human care/wild), family (Otariidae/Phocidae), species, sex (female/male), age (subadult/adult), diet (fresh and/or frozen fish/frozen fish), clinical status at the time of blood collection (apparently healthy/clinically sick), birthplace (Zoomarine/other location), presence of felids around habitats (absent/present), pregnancy (non-pregnant/pregnant) and abortion (none miscarriage/≥1 miscarriage). Data on the control of arthropods and rodents were also recorded. Clinical manifestations included gastrointestinal (diarrhea, nausea, vomiting, abdominal pain), respiratory (dyspnoea, cough, wheezing) or neurological (ataxia, seizures) signs.

### 4.2. Serological Examination

Serum samples were tested for specific IgG antibodies to *T. gondii* with a MAT commercial kit (Toxo-Screen DA^®^, bioMérieux, Lyon, France) according to the manufacturer’s instructions. Sera were assayed at a two-fold serial dilution from 1:25 to 1:3200. Positive and negative controls were supplied with the kit and included in each testing plate.

The results obtained were expressed as an antibody titre, i.e., the reciprocal of the highest dilution at which agglutination (at least half the well’s diameter) was still visible after 5–18 h incubation at room temperature. A cut-off titre of 25 was chosen based on previous studies on pinnipeds [14,16,18,19,20]. Due to its sensitivity and specificity, the MAT is considered the most useful serological test in detecting antibodies to *T. gondii* in animals [1]. The commercial test used in the present study has proven its usefulness in detecting antibodies to *T. gondii* in experimentally [41] and naturally infected pinnipeds [17,24,26,34]. Besides that, this serological method does not require species-specific conjugates and specialized equipment [42,43].

### 4.3. Statistical Analysis

Statistical analysis was performed using the IBM SPSS 26.0 program for Windows. Association between the prevalence of antibodies to *T. gondii* and explanatory variables (provenance, family, sex, age, diet, clinical status, birthplace, presence of felids, gestation and abortion) were analysed using the chi-square or Fisher’s exact tests. The exact binomial test was used to calculate CI for the proportions, with a 95% confidence level. A *p* < 0.05 was defined as statistically significant.

## 5. Conclusions

In conclusion, the results of the present study document that pinnipeds under human care and wild pinnipeds in Portugal are exposed to *T. gondii*. It is noteworthy that infection in captive animals was higher than in wild animals, probably related to exposure to waters contaminated with oocysts excreted by cats. Prevention of access of felids to the enclosure where pinnipeds are housed and contiguous areas, efficient rodent and arthropod control programs, a diet based on previously frozen thawed fish and the use of proper hygiene practices are some of the measures that can minimize the risk of exposure to this parasite. 

## Figures and Tables

**Table 1:** *Toxoplasma gondii* positivity and modified agglutination test (MAT) titres in pinnipeds under human care and wild pinnipeds.

Common Name (*Scientific name*)	No. MAT Positive/No. Tested (*n*)	Antibody Titres								
		<25	25	50	100	200	400	800	1600	≥3200
**Pinnipeds under human care**	12/60	48	4	1	5	0	0	0	1	1
California sea lion (*Zalophus californianus*)	2/12	10	2	0	0	0	0	0	0	0
Grey seal (*Halichoerus grypus*)	0/7	7	0	0	0	0	0	0	0	0
Harp seal (*Pagophilus groenlandicus*)	0/2	2	0	0	0	0	0	0	0	0
Harbor seal (*Phoca vitulina*)	2/13	11	2	0	0	0	0	0	0	0
Ringed seal (*Pusa hispida*)	0/1	1	0	0	0	0	0	0	0	0
South African fur seal (*Arctocephalus pusillus pusillus*)	4/20	16	0	1	1	0	0	0	1	1
South American sea lion (*Otaria flavescens*)	4/5	1	0	0	4	0	0	0	0	0
**Wild pinnipeds**	3/109	106	1	1	0	0	0	0	0	1
Grey seal (*Halichoerus grypus*)	0/4	4	0	0	0	0	0	0	0	0
Harbor seal (*Phoca vitulina*)	2/99	97	0	1	0	0	0	0	0	1
Hooded seal (*Cystophora cristata*)	1/6	5	1	0	0	0	0	0	0	0
**TOTAL**	15/169	154	5	2	5	0	0	0	1	2

**Table 2:** Seroprevalence of *Toxoplasma gondii* in pinnipeds according to the independent variables studied.

Independent Variable	Animals Tested (*n*)	Relative Distribution (%)	MAT-Positive (*n*)	Prevalence (%)	95% CI
**Provenance**					
Under human care	60	35.5	12	20.0	10.8–32.3
Wild	109	64.5	3	2.8	0.6–7.8
**Family**					
Otariidae	37	21.9	10	27.0	13.8–44.1
Phocidae	132	78.1	5	3.8	1.2–8.6
**Diet**					
Fresh and/or frozen fish	109	64.4	3	2.8	0.6–7.8
Frozen fish	60	35.6	12	20.0	10.8–32.3
**Sex**					
Female	70	41.4	7	10.0	4.1–19.5
Male	99	58.6	8	8.1	3.6–15.3
**Age**					
Subadult	21	12.4	1	4.8	0.1–23.8
Adult	148	87.6	14	9.5	5.3–15.4
**Clinical status**					
Apparently healthy	57	81.4	8	14.0	6.3–25.8
Clinically sick	13	18.6	5	38.5	13.9–68.4
Birthplace *					
Zoomarine	10	16.7	1	10.0	0.3–44.5
Other location	50	83.3	11	22.0	11.5–36.0
Presence of felids around habitats *					
Absent	0	0	0	0	ND
Present	60	100	12	20.0	10.8–32.3
Pregnancy *					
Non-pregnant	14	60.9	5	35.7	12.8–64.9
Pregnant	9	39.1	1	11.1	0.3–48.3
Abortion *					
No miscarriage	21	91.3	6	28.6	11.3–52.2
≥1 miscarriage	2	8.7	0	0	0.0–84.2
**TOTAL**	169	100	15	8.9	5.1–14.2

* = only pinnipeds under human care; CI = confidence interval; MAT = modified agglutination test; ND = not determined.

**Table 3:** Antibody titres to *Toxoplasma* *gondii* by the modified agglutination test (MAT) in seropositive pinnipeds under human care sampled twice.

Common Name (*Scientific name*)	2001	2002	2003	2004	2005	2007	2013	2017	2019
California sea lion (*Zalophus californianus*)	<25	-	-	-	25	-	-	-	-
California sea lion	-	-	-	50	25	-	-	-	-
Harbor seal (*Phoca vitulina*)	-	<25	-	-	25	-	-	-	-
Harbor seal	-	<25	-	25	-	-	-	-	-
South African fur seal (*Arctocephalus pusillus pusillus*)	-	-	800	-	50	-	-	-	-
South African fur seal	-	-	-	-	≥3200	100	-	-	-
South African fur seal	-	-	-	-	-	-	-	≥3200	≥3200
South American sea lion (*Otaria flavescens*)	-	-	-	100	100	-	-	-	-
South American sea lion	-	-	<25	-	100	-	-	-	-
South American sea lion	<25	-	-	-	100	-	-	-	-
South American sea lion	-	50	-	-	100	-	-	-	-

**Table 4:** Species and number of pinnipeds tested for *Toxoplasma gondii* IgG according to their provenance.

Common Name (*Scientific name*)	Provenance	Location	No. Animals Tested
**Family Otariidae**			
California sea lion (*Zalophus californianus*)	Human care	Zoomarine Portugal	12
South African fur seal (*Arctocephalus pusillus pusillus*)	Human care	Zoomarine Portugal	20
South American sea lion (*Otaria flavescens*)	Human care	Zoomarine Portugal	5
**Family Phocidae**			
Grey seal (*Halichoerus grypus*)	Human care	Zoomarine Portugal	7
	Wild	South Portuguese coast	4
Harbor seal (*Phoca vitulina*)	Human care	Zoomarine Portugal	13
	Wild	Lorenzensplate, Wadden Sea	99
Harp seal (*Pagophilus groenlandicus*)	Human care	Zoomarine Portugal	2
Hooded seal (*Cystophora cristata*)	Wild	South Portuguese coast	6
Ringed seal (*Pusa hispida*)	Human care	Zoomarine Portugal	1

## Data Availability

Data is contained within the article.
